# Improving patient safety by optimizing the use of nursing human resources

**DOI:** 10.1186/s13012-015-0278-1

**Published:** 2015-06-14

**Authors:** Christian M. Rochefort, David L. Buckeridge, Michal Abrahamowicz

**Affiliations:** School of Nursing, Faculty of Medicine, University of Sherbrooke, Campus Longueuil, 150 Place Charles-LeMoyne, Suite 200, Longueuil, Quebec J4K 0A8 Canada; McGill Clinical and Health Informatics Research Group, McGill University, 1140 Pine Avenue West, Montreal, Quebec H3A 1A3 Canada; Department of Epidemiology, Biostatics and Occupational Health, Faculty of Medicine, McGill University, Purvis Hall, 1020 Pine Avenue West, Montreal, Quebec H3A 1A2 Canada; Division of Clinical Epidemiology, McGill University Health Centre, Ross Pavilion, 687 Pine Avenue West, R4.29, Montreal, Quebec H3A 1A1 Canada

**Keywords:** Nurse staffing, Adverse events, Electronic health record, Acute care hospital, Survival analysis, Longitudinal study

## Abstract

**Background:**

Recent ecological studies have suggested that inadequate nurse staffing may contribute to the incidence of adverse events in acute care hospitals. However, longitudinal studies are needed to further examine these associations and to identify the staffing patterns that are of greatest risk. The aims of this study are to determine if (a) nurse staffing levels are associated with an increased risk of adverse events, (b) the risk of adverse events in relationship to nurse staffing levels is modified by the complexity of patient requirements, and (c) optimal nurse staffing levels can be established.

**Methods/design:**

A dynamic cohort of all adult medical, surgical, and intensive care unit patients admitted between 2010 and 2015 to a Canadian academic health center will be followed during the inpatient and 7-day post-discharge period to assess the occurrence and frequency of adverse events in relationship to antecedent nurse staffing levels. Four potentially preventable adverse events will be measured: (a) hospital-acquired pneumonia, (b) ventilator-associated pneumonia, (c) venous thromboembolism, and (d) in-hospital fall. These events were selected for their high incidence, morbidity and mortality rates, and because they are hypothesized to be related to nurse staffing levels. Adverse events will be ascertained from electronic health record data using validated automated detection algorithms. Patient exposure to nurse staffing will be measured on every shift of the hospitalization using electronic payroll records. To examine the association between nurse staffing levels and the risk of adverse events, four Cox proportional hazards regression models will be used (one for each adverse event), while adjusting for patient characteristics and risk factors of adverse event occurrence. To determine if the association between nurse staffing levels and the occurrence of adverse events is modified by the complexity of patient requirements, interaction terms will be included in the regression models, and their significance assessed. To assess for the presence of optimal nurse staffing levels, flexible nonlinear spline functions will be fitted.

**Discussion:**

This study will likely generate evidence-based information that will assist managers in making the most effective use of scarce nursing resources and in identifying staffing patterns that minimize the risk of adverse events.

**Electronic supplementary material:**

The online version of this article (doi:10.1186/s13012-015-0278-1) contains supplementary material, which is available to authorized users.

## Background

Adverse events (AEs) are injuries caused by medical management rather than by the underlying condition of the patient [1]. AEs affect between 2.9 and 16.6 % of all hospitalized patients, and studies have estimated that 30 to 58 % of all AEs are preventable [2–9]. Preventable AEs are associated with significant morbidity and mortality, with 20 to 25 % of all events resulting in permanent disability or death [1, 6]. Preventable AEs are also very costly, representing between US$17 and US$29 billion in additional health care costs annually [4]. Moreover, human errors have been identified as one of the largest contributors to the occurrence of preventable AEs in acute care hospitals [1, 10].

Nurses are the largest component of the acute care hospital workforce, representing 30 to 40 % of hospital staff, accounting for 25 % of the total acute care hospital operating budget and 44 % of direct care costs [11, 12]. Nurses play an important role in the prevention of AEs, as nursing actions such as the ongoing monitoring of patient conditions are related to better patient outcomes [13, 14]. To accomplish these actions, an adequate number of nurses with the appropriate clinical knowledge and skills are needed. Indeed, recent ecological studies have suggested that understaffing and the use of less qualified nursing staff may be important contributors to the incidence of AEs in acute care hospitals [13–16].

The next step in investigation is to determine the optimal nurse staffing levels (i.e., the optimal numbers of nurses and mixes of skills, education, and experience) that are needed to minimize the risk of AEs. This is especially relevant given that the current shortage of nurses is expected to worsen in the coming years [17] and because nursing managers are implementing a variety of staffing policies—for which little empirical evidence is available—to mitigate the shortage of nurses and maintain the safety of patient care (e.g., using overtime hours, hiring less qualified nursing staff such as nursing assistants or registered practical nurses) [18, 19]. In addition, with the current economic downturn, many hospitals are under pressure to identify less costly staffing plans, thus furthering the routine use of less skilled nursing workers and of overtime hours to meet peak staffing demands [20, 21]. To help decision makers establish safe evidence-based staffing policies, the temporal relationships between nurse staffing levels and the incidence of AEs, the thresholds for safe staffing levels, and the potential modification of these relationships by levels of patient complexity need to be determined by conducting longitudinal studies.

Until recently, the detailed daily patient and staffing data needed to conduct such an investigation were not readily available. With the advent of electronic health records (EHR) and digital capture of payroll and staffing data, an exciting opportunity has emerged to address these important questions in the management of nursing staff in acute care hospitals. Taking advantage of this new opportunity, the objectives of this study are to determine if (a) nurse staffing levels are associated with an increased risk of AEs and if particular patterns of nurse staffing are of greatest risk, (b) the risk of AEs in relationship to nurse staffing levels is modified by the complexity of patient requirements, and (c) optimal nurse staffing levels can be established.

## Literature review

The past two decades have witnessed the publication of a large number of studies examining the relationships between nurse staffing levels and AEs. As a group, these studies have provided ecological evidence that acute care hospitals with higher nurse staffing levels have lower rates of mortality and AEs [13–16]. Moreover, they have suggested that while using overtime hours and less qualified nursing staff may provide acceptable numbers of people at bedside, these staffing strategies are also associated with a higher risk of AEs [21–23]. While these studies have made important contributions to the field, the strength of the evidence they have provided has been challenged [13, 24].

Indeed, most of these studies used cross-sectional designs where the exposure to nurse staffing levels and the occurrence of AEs were measured at the same point in time [25, 26]. Because the temporal relationship between the exposure and the outcome cannot be ascertained from these studies, it is difficult to determine if the occurrence of an AE can truly be attributed to the antecedent exposure to low nurse staffing levels. In addition, most of these studies were multi-institutional investigations that used large national, state, or provincial administrative databases to determine if hospital-level measures of nurse staffing were related to AEs after adjusting for hospital case mix [15, 24]. While this approach can be useful for benchmarking purposes, it entails averaging staffing and AE data over relatively long periods of time (e.g. 1 year), and across all types of units (e.g., internal medicine, intensive care unit) and patients within a given hospital [13]. As a consequence, it has been difficult to translate the results of these studies into specific recommendations regarding the optimal nurse staffing levels for a given patient at a specific point in time during the course of a hospitalization [13, 15].

To move this field forward, there is a need for longitudinal studies conducted at the patient level of analysis [16]. Such studies are required not only to determine if nurse staffing levels are related to AEs but also to identify the particular staffing patterns that are of greatest risk, and the specific staffing thresholds that minimize the risk of AEs given the complexity of patient requirements. This is especially important given that the health care industry has lagged behind other safety-sensitive industries, such as aviation and long-distance trucking, in defining staffing policies that minimize the risk of AEs [13, 20]. As a first step towards defining such policies, novel methods for capturing variations in nurse staffing levels through time have recently been developed.

## Pilot work

### Novel methods for measuring patient exposure to nurse staffing levels

We recently used digitized payroll data to examine how variations in nurse staffing levels through time are related to the use of patient sitters, a type of unlicensed assistive health care provider (UAP) whose function is to provide close surveillance to patients at risk of an AE [27, 28]. Using a nested case-control approach, we found that patients exposed to higher volumes of overtime hours and to less experienced nursing staff during the antecedent exposure period had a higher likelihood of sitter use compared to controls (Table 1).

**Table 1:** Nurse staffing characteristics and adjusted model of their effects on sitter use

Nurse staffing characteristics	Descriptive statistics		Adjusted^a^ model
	Sitter use, *M* (SD) (*N* = 1179)	No sitter use, *M* (SD) (*N* = 4167)	OR (95%CI)*
RN overtime hours (per 15 min. ↑ per patient/shift)	15.6 (21.6)	9.6 (18.0)	1.20 (1.07, 1.35)*
Mean RN work experience (per 5 years ↓)	10.1 (2.1)	10.1 (2.7)	1.30 (1.12, 1.55)*

*RN* registered nurse

*P < .001

^a^Adjusted for patient characteristics (age, gender, Charlson score), risk factors for disruptive behaviors and fall-related injuries, psychotropic drug use during the exposure period, and other nurse staffing characteristics potentially associated with sitter use

In the proposed study, we will build on this prior work to examine the associations between nurse staffing levels and the risk of AEs. One of the original contributions of this study, and a direct extension of our prior work in this area, will be to model the effect of nurse staffing levels on the risk of AEs as a time-varying exposure. This will allow to determine if and how the risk of AEs changes with both the duration of exposure to suboptimal staffing (e.g., extensive overtime use), but also with the intensity of exposure (e.g., low volume of nursing hours per patient). This will facilitate the identification of the particular nurse staffing patterns that are of greatest risk. An important pre-requisite to identifying such patterns is to have access to accurate measures not only of AE occurrence but also of their timing.

### Current methods of AE measurement

In their systematic review of the studies examining the associations between nurse staffing levels and AEs, Kane et al. [15] found that 62 % of the existing studies relied on discharge diagnostic codes to identify AEs, 29 % on manual chart review, and 9 % on incident reporting systems. Discharge diagnostic codes have low sensitivity and positive predictive values (PPV) for identifying AEs [29, 30]. In addition, these codes are not dated with precision (beyond being marked as present or not on admission) [31, 32], a limitation that prevents the elucidation of the temporal relationship between nurse staffing levels and the occurrence of AEs. Accurate timing of AE occurrence is indeed a critical requirement if new knowledge is to be generated on the etiologic contribution of various nurse staffing policies on the incidence of AEs. Manual chart review is a time-consuming, resource-intensive, and costly process [33]. As a consequence, studies using this approach are often smaller in size and underpowered for adequately examining how changes in nurse staffing levels relate to the occurrence of AEs [34]. Incident reports are known to underestimate the true incidence of AEs by a factor of about 20 [35, 36].

In sum, the limitations in existing methods for measuring AEs have curtailed the ability to pursue important investigational work on the identification of nurse staffing policies that minimize the risk of AEs. To address these limitations, novel methods of AE detection have recently been developed and validated.

### Novel methods of AE detection

With the increasing availability of rich clinical narrative documents in an electronic format (e.g., radiology reports, progress notes), researchers have started to use automated methods, such as statistical natural language processing (NLP) techniques, to identify AEs [33, 37]. Statistical NLP techniques use probabilistic approaches to automatically classify a set of documents into one of a discrete set of predefined categories (e.g., positive or negative for pneumonia) [38]. Prior studies have shown that statistical NLP can be highly accurate for identifying AEs.

For instance, we recently conducted a pilot study to validate the accuracy of using statistical NLP for identifying venous thromboembolism (VTE), an AE that includes both deep vein thrombosis (DVT) and pulmonary embolism (PE), from electronic narrative radiology reports [39]. The statistical NLP model predicting DVT achieved a sensitivity of 0.80 (95 %CI 0.76–0.85), a specificity of 0.98 (95 %CI 0.97–0.99), and a PPV of 0.89 (95 %CI 0.85–0.93). As for the statistical NLP model predicting PE, sensitivity was 0.79 (95 %CI 0.73–0.85), specificity 0.99 (95 %CI 0.98–0.99), and PPV 0.84 (95 %CI 0.75–0.92) [39]. Since then, we have received funding from the Canadian Institutes of Health Research to adapt this technique for the detection of other AE indicators, including hospital-acquired pneumonia (HAP), ventilator-associated pneumonia (VAP), and in-hospital falls. Preliminary studies using NLP-based approaches for detecting these AEs have shown good prediction performances, with sensitivities ranging from 83 to 86 %, PPV ranging from 85 to 100 %, and specificities ranging from 90 to 100 % [40, 41].

An important advantage of statistical NLP, in addition to being highly accurate, is that large amounts of EHR data can be scanned with minimal human effort and cost [33], a major gain compared to using manual chart review. Moreover, because free-text radiology reports are dated, the timing of AE occurrence can be determined with relatively high precision, a net improvement compared to using discharge diagnostic codes. In the proposed study, we will take advantage of these characteristics and build on our prior research work to assess the temporality of the associations between nurse staffing levels and the risk of AEs.

## Methods

### Setting

This study will be conducted at the McGill University Health Centre (MUHC), an academic health center located in the Canadian province of Quebec. The MUHC is composed of five adult care hospitals and has more than 800 beds. It receives close to 40,000 inpatient admissions and performs more than 34,000 surgeries yearly. The MUHC has more than 11,500 employees, including 3000 registered nurses (RNs) and nursing assistants (NAs), and 800 patient care assistants (PCAs) [42].

### Study design and population

**Fig. 1:**
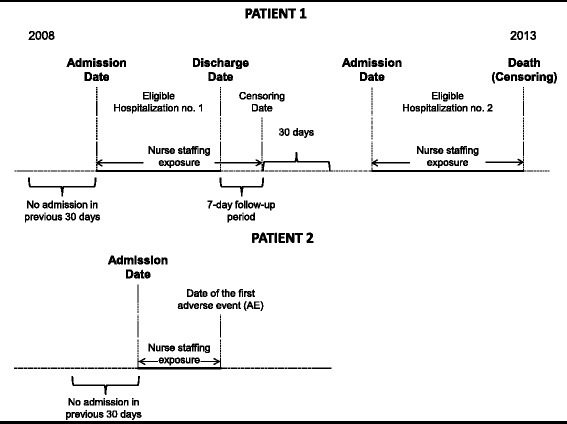
Study design

A dynamic cohort of all adult medical, surgical, and intensive care unit (ICU) patients admitted to the MUHC between January 1, 2010, and December 31, 2015, will be followed during the inpatient and 7-day post-discharge period to assess the occurrence and frequency of four AEs (i.e., VTE, HAP, VAP, and in-hospital falls) in relationship to antecedent nurse staffing levels. A follow-up period of 7 days post-discharge will be observed to allow enough time for patients with an AE “incubating” at the time of discharge (e.g., HAP, VTE) to develop the symptoms of the disease and return to the hospital (Fig. 1) [43]. Because in-hospital falls, by definition, cannot occur after discharge, the follow-up period for this particular AE will stop at hospital discharge [43]. For VAP, only the subset of patients who will be intubated at any point over the course of a hospitalization will be considered at risk of developing the disease. Patients will be enrolled in the cohort if they were (a) admitted on a medical, surgical, or ICU at the MUHC; (b) not admitted for one of the AEs of interest; and (c) not hospitalized in the previous 30 days. Re-hospitalization by the same patients occurring more than 30 days after the end of the follow-up period for a given hospitalization will be eligible for inclusion (Fig. 1) [43, 44].

AEs will be ascertained by applying statistical NLP models to EHR data retrieved at the MUHC. For patients experiencing multiple AEs, or relapses of the same AE over a given hospitalization, only the first AE will be selected. AEs occurring during the follow-up period will be ascertained from EHR data at the time of hospital readmission or emergency department (ED) visit (approximately 9 % of patients return to the ED within 7 days of discharge; 86 % to the same hospital from which they were discharged) [45]. Patients (a) with no AE until the end of the follow-up, (b) readmitted to the hospital during the follow-up period for any reason other than the AEs of interest, or (c) who die before experiencing an AE will be censored at that time (Fig. 1). Patients will be assumed to survive throughout the 7-day post-discharge period.

Nurse staffing levels for a given patient will be determined for each shift of a hospitalization using electronic payroll records. The association between nurse staffing levels and the risk of AEs will be assessed using survival analysis with time-dependent measures of nurse staffing, patient complexity as a confounder and potential modifier of the association, and patient hospitalization as the unit of analysis for AE assessment.

### Data sources

Data required for this study will be extracted from the MUHC clinical data warehouse and will be linked by unit, patient, and hospital admission date and time. Specifically, 12 electronic databases will be queried, including (1) laboratory and microbiology; (2) radiology; (3) vital signs; (4) ICU; (5) pharmacy; (6) admission, discharge, and transfer; (8) discharge abstracts; (9) emergency; (10) operating room; (11) incident reports; and (12) payroll.

### Measures

#### Study outcomes: AE indicators

Four AE indicators will be measured: (a) HAP, defined as a pulmonary infection that occurs 48 h or more after hospital admission in patients with no evidence of pneumonia at the time of hospital admission [46]; (b) VAP, defined as a pulmonary infection occurring more than 48 h after endotracheal intubation and mechanical ventilation in patients with no evidence of pneumonia at the time of intubation [46] (pneumonia will be considered as a VAP up until 72 h after extubation) [47]; (c) VTE, defined as a DVT of the lower or upper extremities or a PE occurring 48 h or more after hospital admission [48]; and (d) in-hospital falls, defined as an unplanned descent to the floor with or without injury occurring during the course of a hospitalization [49].

These AEs were selected because their occurrence is hypothesized to be related to nurse staffing levels [13–16] and because they can result in significant morbidity, mortality, and cost [49–52]. Moreover, they all have high incidence rates compared to other AEs. Indeed, HAP represents one of the most common nosocomial infections, accounting for 15 % of all hospital-acquired infections and 25 % of all ICU-acquired infections [46, 53, 54]. HAPs are estimated to occur at a rate of between 5 and 20 cases per 1000 hospital admissions [54]. VAP represents the most frequent ICU-acquired infection, occurring in 9 to 28 % of patients receiving mechanical ventilation [54–57]. In the absence of thromboprophylaxis, the incidence of VTE ranges from 10 to 40 % in the medical and general surgical populations to as high as 40 to 60 % in patients who have undergone major orthopedic surgical procedures [58, 59]. Falls are estimated to occur in 1.9 to 3 % of all hospitalizations and in 2 to 27 % of elderly hospitalizations [49].

AE occurrence will be ascertained by applying statistical NLP models to electronic narrative radiological and incident reports retrieved at the MUHC [39–41]. These models have learned from human-labeled training documents which combinations of words in narrative reports are suggestive of the presence of a given AE. The accuracy of these models is summarized in Table 2. For all patients, the date and time of the narrative report will be considered as the date and time of AE occurrence.

**Table 2:** Accuracy of the automated AE detection algorithms

Accuracy measures	VTE [39]		Pneumonia [40]	In-hospital fall [41]
	DVT	PE		
	Coefficient (95%CI)	Coefficient (95%CI)	Coefficient (95%CI)	Coefficient (95%CI)
Sensitivity (%)	80 (76–85)	79 (73–85)	83 (80–86)	83 (81–85)
Specificity (%)	98 (97–99)	99 (98–99)	98 (97–99)	100 (100–100)
PPV (%)	89 (85–93)	84 (75–92)	89 (84–93)	100 (100–100)
NPV (%)	96 (93–99)	98 (98–99)	97 (96–97)	99 (98–99)

*DVT* deep vein thrombosis, *NPV* negative predictive value, *PE* pulmonary embolism, *PPV* positive predictive values, *VTE* venous thromboembolism

### Nurse staffing levels

#### Primary attributes of nurse staffing

Nurse staffing levels in a given hospital typically vary from one unit to the next as well as within a given unit on a shift-by-shift basis as a function of the fluctuations in patients’ requirements for nursing care, as well as with unpredictable absenteeism [24]. For this reason, patient exposure to nurse staffing levels will be measured on every shift of a hospitalization using the following three time-varying indicators:

*Nurse staffing intensity* will be defined as the average number of work hours per patient provided by all members of the nursing staff (i.e., RNs, NAs, PCAs, and UAPs) [60, 61]. For each patient, nurse staffing intensity will be measured by dividing the total number of nursing work hours on the unit where the patient was hospitalized on a given shift by the beginning-of-shift patient census on that unit for that shift [61].

*Skill mix* will be defined as the proportion of the total nursing work hours that were reported by RNs [61]. For each patient, skill mix will be measured by dividing the total number of RN work hours on the unit where the patient was hospitalized on a given shift by the total number of nursing work hours on that unit for that shift.

*Overtime use* will be defined as time worked in excess of the standard hours and paid at least one and one-half (1.5) times the regular rate of pay [19, 21]. For each patient, overtime use will be measured by dividing the total number of overtime hours reported by all members of the nursing staff on the unit where the patient was hospitalized on a given shift by the total number of nursing work hours reported on that unit for that shift.

#### Secondary attributes of nurse staffing

Other nurse staffing attributes can potentially be associated with AE occurrence, including nursing staff levels of experience and education [60, 62]. Patient exposure to these staffing attributes will be measured on every shift of a given hospitalization using the following time-varying indicators:

*Experience* is expected to provide nurses with exposure to different patient conditions and clinical scenarios which contribute to the development of their knowledge, skills, and critical thinking [63, 64]. More experienced nurses should thus have a broader set of interventions to prevent AEs from occurring. Conversely, when the overall level of experience in a team of nurses is reduced, more AEs may occur [28]. For each patient, nurse experience will be measured as the mean number of years of experience held by all members of the nursing staff who reported work hours on the unit where the patient was hospitalized on a given shift [61].

*Education* (at the baccalaureate degree or higher) is thought to be associated with better critical thinking and clinical judgment skills among RNs, and ecological studies have suggested that hospitals with more highly educated RNs have lower rates of AEs and mortality [34, 64]. For each patient, education will be measured as the proportion of RNs with a baccalaureate degree or higher among all RNs who reported work hours on the unit where the patient was hospitalized on a given shift [61].

### Duration and intensity of exposure to nurse staffing

Time-varying exposure to nurse staffing levels will be measured for every shift of a given hospitalization. Four alternative measures will be used to represent nurse staffing levels: (a) *current exposure*, defined as nurse staffing levels on the current shift; (b) *mean recent exposure*, defined as mean staffing levels over the past 4 days; (c) *mean exposure* since hospital admission, defined as mean staffing levels since hospital admission, and (d) *weighted cumulative exposure* (WCE) [65]. WCE is a novel analytical method, developed and validated in our prior research work [65, 66], which adds the dimension of timing of past exposures, along with information about duration and intensity, into a single metric. This metric is created by multiplying each exposure in the past by a weight that represents the relative importance of that exposure on the current risk of an AE, and summing the weighted past exposures up to the current time [65]. The weighting function that assigns weights to past exposures is estimated directly from the data [65]. This novel approach should provide additional insights about the mechanism by which time-dependent patterns of nurse staffing influence the risk of AEs. Because no data on nurse staffing levels during the post-discharge follow-up period will be available, a sensitivity analysis will be conducted to determine which, among several alternatives, provides the best estimates for each measure of staffing (e.g., assigning average expected nurse staffing values based on the patient’s destination upon discharge, carrying over the last nurse staffing values at discharge, setting all nurse staffing values to 0).

The aforementioned measures of nurse staffing exposures were selected based on the following hypotheses: For HAP and VAP, suboptimal staffing levels have been shown to result in lapses in basic infection control measures (e.g., hand washing) which may facilitate the transmission of pathogens [67, 68]. Given that the typical incubation period for HAP and VAP is at least 48 h [46], recent exposure to suboptimal nurse staffing levels (e.g., 4 days before the event) may be the most relevant for HAP and VAP. Prolonged immobility has been identified as an important risk factor for VTE [69], and studies have shown that certain thromboprophylaxis measures, such as patient mobilization, are performed less frequently when staffing levels are suboptimal [70]. As such, sustained exposure to suboptimal staffing during a hospitalization may result in a greater risk of VTEs. Lastly, suboptimal staffing levels may also reduce nurses’ capacity to provide adequate surveillance to patients at risk of falling [64, 71]. Because only a small fraction of time is needed for a fall to occur, it is likely that reduced surveillance capacity resulting from suboptimal staffing levels on a given shift would be related to a greater risk of in-hospital falls [71].

### Potential confounders common to all four AE indicators

Several patient and organizational characteristics may increase the likelihood of HAP, VAP, VTE, or in-hospital fall. These conditions will be measured as potential confounders.

*Patient demographic characteristics and comorbidities* are important modifiers of a patient’s health condition and have been associated with an increased risk of AEs [50, 57, 58]. Patient age at hospital admission and sex will be measured as fixed-in-time covariates. Comorbidities will be measured using the Charlson Comorbidity Index, a weighted index of 17 comorbidities that are associated with an increased risk of mortality [72, 73]. Comorbidities will be measured at the time of hospital admission as a fixed-in-time covariate using discharge diagnostic codes from all prior hospitalizations since 2004 (i.e., the maximum time frame for which complete data from previous hospitalizations are available).

*Severity of illness* may influence the risk of AEs and nurse staffing levels [24]. To adjust for this possible source of confounding, severity of illness will be measured as a time-varying covariate using the laboratory-based acute physiology score (LAPS) [74, 75]. The LAPS is a scoring system that integrates information from 14 laboratory tests into a single continuous variable. LAPS will be measured at the beginning of every shift using the most recent laboratory results. LAPS can range from 0 (corresponding to lab results within normal physiologic ranges) to a theoretical maximum of 256. A score of 0 points will be assigned to any unperformed laboratory test [74, 75].

*Admission diagnosis* may increase the likelihood of AEs and will be measured at the time of hospital admission as a fixed-in-time covariate [50, 57, 58]. Admission diagnoses will be classified into 44 categories using a procedure based on diagnostic codes [74].

*Patient turnover rates*, defined as the number of admissions, discharges, and transfers on a given shift [24], may increase the demands on the nursing staff and result in errors or lapses in care processes that may increase the risk of AEs [76, 77]. Patient turnover rates will be measured as a time-varying covariate representing the total number of admissions, transfers, and discharges on the unit where the patient was hospitalized on a given shift divided by the start-of-shift patient census on that unit for that shift.

*The type of nursing unit* where the patient is located may influence both the risk of AE and nurse staffing levels [60]. To adjust for possible confounding from measures of staffing and hospitalization on certain units (e.g., ICU vs. medicine), and for other unmeasured unit-level factors that may increase the risk of AEs (e.g., work environment characteristics) [60], a time-varying covariate representing the nursing unit where the patient is currently hospitalized will be created and updated every shift.

*Other confounders* include three additional fixed-in-time covariates which will be measured at the time of hospital admission: the hospital at which the patient was admitted (to test for differences between the five MUHC hospitals), the year, and the month in which the admission took place (to test for temporal and seasonal trends).

### Potential confounders specific to each AE indicator

In addition to confounders common to all four AEs, AE-specific confounders will also be considered:

*VAP/HAP*: (a) *Length of mechanical ventilation* will be measured at the beginning of every shift as the cumulative number of shifts for which the patient is under mechanical ventilation (cumulative use) [46]. (b) *Recent surgeries* will be measured every shift as a time-varying covariate indicating whether or not the patient had a surgical procedure [54]. In addition, the type of the latest surgical procedure performed (i.e., cardiothoracic, orthopedic, abdominal, other, none) will also be captured by a categorical covariate updated every shift [53, 54]. (c) *Drugs associated with an increased likelihood of pneumonia* will be grouped in two therapeutic classes based on the American Hospital Formulary Service (AHFS) classification [78]: (i) corticosteroids and (ii) gastro-protective agents [53, 54]. The usage of these drugs will be assessed as two time-varying covariates representing current use (yes or no) and will be updated every shift.

*In-hospital fall*: (a) *Gait and mobility impairments* will be measured as a fixed-in-time covariate at the time of hospital admission [50]. Validated coding rules will be used to identify discharge diagnostic codes from all prior hospitalizations since 2004 that are suggestive of the presence of gait and mobility impairments [79]. (b) *Cognitive impairments* will be measured as a fixed-in-time covariate at the time of hospital admission [50]. Coding rules from the Dementia, Delirium and Other Cognitive Impairments subclassification of the Mental Health and Substance Abuse Clinical Classification will be used to identify discharge diagnostic codes from all prior hospitalizations since 2004 that are suggestive of the presence of cognitive impairments [80]. (c) *History of previous falls* will be measured at the time of hospital admission as a fixed-in-time dichotomous covariate (yes or no) [50]. Patients with a history of previous falls will be identified by applying our statistical NLP model for inpatient falls to radiological and incident reports from prior hospitalizations since 2004 [41]. (d) *Psychoactive drugs associated with an increased risk of falling* will be grouped into six therapeutic classes based on the AHFS classification [78]: (i) anticonvulsants; (ii) antidepressants; (iii) antipsychotics; (iv) anxiolytics, sedatives, and hypnotics; (v) benzodiazepines; and (vi) opioids. Psychoactive drug use will be assessed as six time-varying covariates representing current use (yes or no) and will be updated every shift [50].

*VTE*: (a) *History of VTE* will be measured at the time of hospital admission as a fixed-in-time covariate indicating whether or not the patient had a VTE in the past [58]. History of VTE will be determined by applying our statistical NLP models to narrative reports of radiological examinations performed since 2004 [39]. (b) *Anticoagulant drug use* will be assessed as a time-varying covariate updated every day and representing current use (yes or no). (c) *Recent surgeries* will be measured using the approach described for VAP. (d) *Mobility impairments* will be measured using the approach described for in-hospital fall [58].

### Statistical analyses

Descriptive statistics will be used to summarize patient and nurse staffing characteristics. To examine the associations between nurse staffing levels and the risk of HAP, VAP, VTE, and in-hospital falls, four separate AE-specific Cox proportional hazards regression models will be used (i.e., one model for each AE indicator). Time 0 will correspond to the date of hospital admission, and the time to event will be defined as the time to the first AE specific to each of the four models. Patients who had no AE by the end of the follow-up period for a hospitalization episode or who die before experiencing an AE will be censored at that time. All models will be adjusted for patient demographic characteristics, comorbidities, and AE-specific risk factors. For continuous covariates, we will use the flexible fractional polynomials approach to test for nonlinear effects and, if necessary, account for such nonlinearities [81].

Within each regression model, exposure to primary nurse staffing attributes (i.e., staffing intensity, skill mix, and overtime) and secondary nurse staffing attributes (i.e., education and experience) will be defined in four alternative ways, each using a different time-varying exposure or time window: (a) current exposure, (b) mean recent exposure over the past 4 days, (c) mean exposure since hospital admission, and (d) WCE [65]. For WCE, the weights assigned to past exposures will be estimated using a flexible cubic spline function to avoid any a priori assumptions regarding the shape of the weight function [65, 82]. Each model will be adjusted for both fixed (e.g., age, sex) and time-varying covariates (e.g., severity of illness, medication use) relevant to the AE indicator being considered. The fit of the alternative exposure models will be compared using the Akaike information criterion (AIC) [83], and the best-fitting (minimum AIC) model for a given AE indicator will be selected for subsequent analyses [66]. Hazard ratios (HRs) and 95 % confidence intervals (95 %CIs) will be estimated for the best fitting models. To assess if the association between nurse staffing levels and AEs is modified by the complexity of patient requirements, the significance of interaction terms between nurse staffing attributes and selected covariates (e.g., patient type [medical vs. surgical vs. ICU], turnover rates) will be tested. Stratified models will be estimated for each modifying effect.

To assess for the presence of optimal nurse staffing thresholds, for each staffing-by-complexity stratum, a flexible extension of the Cox model that uses nonlinear spline functions to estimate how the hazard varies with increasing value of the predictor (here, nurse staffing levels) will be fitted [82]. The null hypothesis that the effect of nurse staffing levels is linear will be tested with a nonparametric likelihood ratio test (LRT), comparing the partial deviance of the best-fitting linear models with that of the nonlinear spline model [82]. *P* < .05 for the LRT will indicate that the nonlinear model provides significantly better prediction of AEs than the linear model, in which case spline functions may indicate the threshold effect of nurse staffing [84]. The HRs and the 95 %CI for nonlinear HRs derived from splines will be estimated using bootstrap resampling [85].

The proportional hazards (PHs) assumption will be verified with a nonparametric LRT comparing partial deviance of the conventional PH to a flexible non-PH model [82]. In the case of significant violation of the PH hypothesis, the flexible model will estimate how the covariate effect (adjusted HR) changes during the follow-up [82]. To account for possible nonrandom (informative) censoring on death, inverse probability censoring weights will be used in sensitivity analyses [86]. To account for data clustering resulting from repeated hospitalizations by the same patient through time, the marginal approach with robust standard error estimators will be used [87]. Cox regression will be implemented with SAS, version 9.2, and flexible spline-based models [82], and the WCE model [65] will be implemented with customized programs in R.

#### Sample size requirements

We estimate that the Cox regression models for the current exposure to each of the three main nurse staffing attributes will have excellent 90 % power (at two-tailed alpha = 0.05) to detect reductions in the risk of HAP or VTE of (a) 3 to 5 % for every increase of 10 min per patient per shift in nurse staffing intensity and (b) 4 to 6 % for every 5 % increase per shift in skill mix. In addition, this model will have 90 % power to detect a risk increase of 5 to 7 % for every 10 additional minutes per patient per shift of overtime (Additional file 1: Tables A1 and A2). Given that in-hospital falls and VAP have higher incidence rates than HAP and VTE, the detectable HRs for these AEs are smaller (Additional file 1: Tables A3 and A4).

## Discussion

### Current study status

At the present time, we have received both research ethics and institutional approvals to begin the study and extract the required data. Data extraction is expected to begin shortly.

### Relevance and impact

This study will be the first to model the effect of nurse staffing strategies on the risk of AEs as time-varying exposures. This will allow determining if and how the risk of AEs changes with both the duration of exposure to suboptimal staffing (e.g., extensive overtime use), but also with the intensity of exposure (e.g., low volume of nursing hours per patient). This will facilitate the identification of the particular nurse staffing patterns that are of greatest risk. This study will likely generate evidence-based information that will assist managers in making the most effective use of scarce nursing resources and in identifying staffing patterns that minimize the risk of AEs.

## Additional file

Additional file 1:
**Details of sample size calculation.**

## Abbreviations

AE: Adverse event
AIC: Akaike information criterion
DVT: Deep vein thrombosis
ED: Emergency department
EHR: Electronic health records
HAP: Hospital-acquired pneumonia
HR: Hazard ratio
ICU: Intensive care unit
LAPS: Laboratory acute physiology score
LRT: Likelihood ratio test
MUHC: McGill University Health Centre
NA: Nursing assistant
NLP: Natural language processing
PCA: Patient care assistants
PE: Pulmonary embolism
PH: Proportional hazard
PPV: Positive predictive values
RN: Registered nurse
RPN: Registered practical nurse
UAP: Unlicensed assistive health care providers
VAP: Ventilator-associated pneumonia
VTE: Venous thromboembolism
WCE: Weighted cumulative exposure
