# Supplementary material for: Improving patient safety by optimizing the use of nursing human resources
Source: Implement Sci. 2015 Jun 14;10:89. doi: 10.1186/s13012-015-0278-1 (PMC4465738; doi:10.1186/s13012-015-0278-1)
Supplement: Additional file 1: — Details of sample size calculation. [file 13012_2015_278_MOESM1_ESM.docx]

**Details of sample size calculations**

Assumptions:

- Based on our prior work at the McGill University Health Centre (MUHC),[1-3] it is expected that approximately 20,000 hospitalizations per year will be eligible. Of these, approximately 12% will be intensive care unit (ICU) admissions who received mechanical ventilation. Over the 5-year follow-up period, this will provide us with a sample size of at least:
  - 100,000 hospitalizations for the analyses pertaining to hospital-acquired pneumonia (HAP), venous thromboembolism (VTE), and in-hospital fall;
  - 12,000 hospitalizations for the analyses pertaining to ventilator-associated pneumonia (VAP);
- Based on the literature, the incidence rates of the four adverse events (AEs) selected should range between:
  - HAP: 1.0% to 2.0%;[4;5]
  - VTE: 1.0% to 2.0%;[6;7]
  - In-hospital fall: 1.9% to 3.0%;[8;9]
  - VAP: 8.0% to 28.0%;[10;11]
- Based on our previous work,[1;2] the standard deviations (SDs) for each of the three main nurse staffing exposure variables are expected to be within the following ranges:
  - Staffing intensity: 22 to 35 minutes per patient per shift;
  - Skill mix: 5 to 10% per shift;
  - Overtime: 18 to 22 minutes per patient per shift;

Based on these assumptions, the hazard ratios (HRs) that can be detected with 80% and 90% power are presented in Table A1 to Table A4, for VTE, HAP, in-hospital fall and VAP, respectively. These HRs are for Cox regression models that assess the association between the current nurse staffing levels and the risk of each of the four outcomes. All calculations were performed for two-sided tests of significance with alpha=0.05. The influence of the confounder variables on the sample size calculations was accounted for by assuming R^2^=0.2 for the exposition variables with the other covariates. The calculations were made with the software PASS (Hintze J. [2008], PASS 2008, version 8.05. NCSS, LLC. Kaysville, Utah. www.ncss.com).

| **Tables A1**. Hospital-acquired pneumonia (HAP) **-** Detectable hazard ratios for the main nurse staffing exposures | | | | | |
| --- | --- | --- | --- | --- | --- |
|  |  | **Detectable hazard ratios (HRs)** | | | |
| **Nurse staffing levels** | Standard deviations (SDs) | 80% power | | 90% power | |
|  |  | Proportion of events | | Proportion of events | |
|  |  | 1.0% | 2.0% | 1.0% | 2.0% |
| Intensity (per 10 minutes ↑ per patient per shift) | SD = 22 | 0.96 | 0.97 | 0.95 | 0.96 |
|  | SD = 35 | 0.97 | 0.98 | 0.97 | 0.98 |
| Skill mix (per 5% ↑ per shift) | SD = 10 | 0.95 | 0.97 | 0.94 | 0.96 |
|  | SD = 15 | 0.97 | 0.98 | 0.96 | 0.97 |
| Overtime (per 10 minutes ↓ per patient per shift) | SD = 18 | 1.06 | 1.04 | 1.07 | 1.05 |
|  | SD = 22 | 1.05 | 1.03 | 1.05 | 1.04 |

| **Tables A2**. Venous thromboembolism (VTE) **-** Detectable hazard ratios for the main nurse staffing exposures | | | | | |
| --- | --- | --- | --- | --- | --- |
|  |  | **Detectable hazard ratios (HRs)** | | | |
| **Nurse staffing levels** | Standard deviations (SDs) | 80% power | | 90% power | |
|  |  | Proportion of events | | Proportion of events | |
|  |  | 1.0% | 2.0% | 1.0% | 2.0% |
| Intensity (per 10 minutes ↑ per patient per shift) | SD = 22 | 0.96 | 0.97 | 0.95 | 0.96 |
|  | SD = 35 | 0.97 | 0.98 | 0.97 | 0.98 |
| Skill mix (per 5% ↑ per shift) | SD = 10 | 0.95 | 0.97 | 0.94 | 0.96 |
|  | SD = 15 | 0.97 | 0.98 | 0.96 | 0.97 |
| Overtime (per 10 minutes ↓ per patient per shift) | SD = 18 | 1.06 | 1.04 | 1.07 | 1.05 |
|  | SD = 22 | 1.05 | 1.03 | 1.05 | 1.04 |

| **Tables A3**. In-hospital fall - Detectable hazard ratios for the main nurse staffing exposures | | | | | |
| --- | --- | --- | --- | --- | --- |
|  |  | **Detectable hazard ratios (HRs)** | | | |
| **Nurse staffing levels** | Standard deviations (SDs) | 80% power | | 90% power | |
|  |  | Proportion of events | | Proportion of events | |
|  |  | 1.9% | 3.0% | 1.9% | 3.0% |
| Intensity (per 10 minutes ↑ per patient per shift) | SD = 22 | 0.97 | 0.97 | 0.96 | 0.97 |
|  | SD = 35 | 0.98 | 0.98 | 0.98 | 0.98 |
| Skill mix (per 5% ↑ per shift) | SD = 10 | 0.96 | 0.97 | 0.96 | 0.97 |
|  | SD = 15 | 0.98 | 0.98 | 0.97 | 0.98 |
| Overtime (per 10 minutes ↓ per patient per shift) | SD = 18 | 1.04 | 1.03 | 1.05 | 1.04 |
|  | SD = 22 | 1.03 | 1.03 | 1.04 | 1.03 |

| **Table A4.** Ventilator associated pneumonia (VAP) - Detectable hazard ratios for the main staffing exposures | | | | | |
| --- | --- | --- | --- | --- | --- |
|  |  | **Detectable hazard ratios (HRs)** | | | |
| **Nurse staffing levels** | Standard deviations (SDs) | 80% power | | 90% power | |
|  |  | Proportion of events | | Proportion of events | |
|  |  | 8% | 28% | 8% | 28% |
| Intensity (per 10 minutes ↑ per patient per shift) | SD = 60 | 0.98 | 0.99 | 0.98 | 0.99 |
|  | SD = 84 | 0.99 | 0.99 | 0.99 | 0.99 |
| Skill mix (per 5% ↑ per shift) | SD = 6 | 0.92 | 0.96 | 0.91 | 0.95 |
|  | SD = 10 | 0.95 | 0.97 | 0.94 | 0.97 |
| Overtime (per 10 minutes ↓ per patient per shift) | SD = 18 | 1.06 | 1.03 | 1.07 | 1.04 |
|  | SD = 32 | 1.03 | 1.02 | 1.04 | 1.02 |
| Note: In the intensive care unit (ICU), the standard deviation (SD) for staffing intensity is expected to range between 60-84 minutes per patient per shift, whereas the SD for overtime is expected to range between 18-32 minutes per patient per shift. | | | | | |

Reference List

1. Rochefort CM, Ward L, Ritchie JA, Girard N, Tamblyn RM: **Patient and nurse staffing characteristics associated with high sitter use costs**. *J Adv Nurs* 2012, **68**(8):1758-67.

2. Rochefort CM, Ward L, Ritchie JA, Girard N, Tamblyn RM: **Registered nurses' job demands in relation to sitter use: nested case-control study**. *Nurs Res* 2011, **60**(4):221-30.

3. Rochefort CM, Buckeridge DL, Fragos J, Jauvin C, Tamblyn R: **Accuracy of an automated method for detecting cases of deep vein thrombosis from electronic radiological reports.** *JAMIA* 2013.

4. Chastre J, Fagon JY: **Ventilator-associated pneumonia**. *Am J Respir Crit Care Med* 2002, **165**(7):867-903.

5. American Thoracic Society and Infection Disease Society of America: **Guidelines for the management of adults with hospital-acquired, ventilator-associated, and healthcare-associated pneumonia**. *Am J Respir Crit Care Med* 2005, **171**(4):388-416.

6. Cracowski JL, Bosson JL, Baloul F, Moirant C, Hunt M, Merloz P, Carpentier P, Franco A: **Early development of deep-vein thrombosis following hip fracture surgery: the role of venous wall thickening detected by B-mode ultrasonography**. *Vasc Med* 1998, **3**(4):269-74.

7. Fanikos J, Rao A, Seger AC, Piazza G, Catapane E, Chen X, Goldhaber SZ: **Venous thromboembolism prophylaxis for medical service-mostly cancer-patients at hospital discharge**. *Am J Med* 2011, **124**(12):1143-50.

8. Currie L: **Fall and Injury Prevention**. In *Patient Safety and Quality: An Evidence-Based Handbook for Nurses.*Hughes RG. Rockville (MD): Agency for Healthcare Research and Quality; 2008.

9. Salgado RI, Lord SR, Ehrlich F, Janji N, Rahman A: **Predictors of falling in elderly hospital patients**. *Arch Gerontol Geriatr* 2004, **38**(3):213-9.

10. Patel PJ, Leeper KV, Jr., McGowan JE, Jr.: **Epidemiology and microbiology of hospital-acquired pneumonia**. *Semin Respir Crit Care Med* 2002, **23**(5):415-25.

11. Myrianthefs PM, Kalafati M, Samara I, Baltopoulos GJ: **Nosocomial pneumonia**. *Crit Care Nurs Q* 2004, **27**(3):241-57.
